# May I Cut in? Gene Editing Approaches in Human Induced Pluripotent Stem Cells

**DOI:** 10.3390/cells6010005

**Published:** 2017-02-06

**Authors:** Nicholas Brookhouser, Sreedevi Raman, Christopher Potts, David. A. Brafman

**Affiliations:** School of Biological and Health Systems Engineering, Arizona State University, 501 E. Tyler Mall, ECG 334A, Tempe, AZ 85287, USA; brookhouser.nick@gmail.com (N.B.); sraman13@asu.edu (S.R.); pottsc13@gmail.com (C.P.)

**Keywords:** human induced pluripotent stem cells (hiPSCs), genome editing, homology-directed repair, ZFN, TALEN, CRISPR/Cas9

## Abstract

In the decade since Yamanaka and colleagues described methods to reprogram somatic cells into a pluripotent state, human induced pluripotent stem cells (hiPSCs) have demonstrated tremendous promise in numerous disease modeling, drug discovery, and regenerative medicine applications. More recently, the development and refinement of advanced gene transduction and editing technologies have further accelerated the potential of hiPSCs. In this review, we discuss the various gene editing technologies that are being implemented with hiPSCs. Specifically, we describe the emergence of technologies including zinc-finger nuclease (ZFN), transcription activator-like effector nuclease (TALEN), and clustered regularly interspaced short palindromic repeats (CRISPR)/Cas9 that can be used to edit the genome at precise locations, and discuss the strengths and weaknesses of each of these technologies. In addition, we present the current applications of these technologies in elucidating the mechanisms of human development and disease, developing novel and effective therapeutic molecules, and engineering cell-based therapies. Finally, we discuss the emerging technological advances in targeted gene editing methods.

## 1. Introduction

The development of methods for the reprogramming of terminally differentiated somatic cells (e.g., skin fibroblasts, peripheral blood mononuclear cells) to a pluripotent state has provided new opportunities to study complex diseases in a simplified and accessible system as well as generate mature cell populations for regenerative medicine-based therapies. Since the initial reports of Yamanaka and colleagues generating induced pluripotent stem cells (iPSCs) from mouse [1] and subsequently human [2] cells by the ectopic expression of four transcription factors—KLF4, OCT4, SOX2, and cMYC—several improvements to iPSC generation methods have been reported including (i) generation of hiPSCs in the absence of the oncogene cMYC [3], (ii) development of ‘foot-print free’ episomal- [4], mRNA- [5], protein- [6], and chemical-based [7] reprograming methods that do not require genomic integration of the reprogramming factors, (iii) improvements in reprogramming efficiency through the use of small molecules [8,9] and microRNAs [10], and (iv) derivation and culture of hiPSC in completely defined, xeno-free conditions [11]. These technological advances have allowed for the development of hiPSC-based models of numerous neurodegenerative [12,13,14,15,16], psychiatric [17], cardiovascular [18,19], renal [20], pulmonary [21,22], hepatic [23], metabolic [24,25], and mitochondrial [26,27] diseases and disorders. In addition, progress in cellular reprogramming methods have allowed for further developments in therapies aimed to repair or replace diseased and dysfunctional cells in vivo [28]. In fact, in September 2014, Takahashi and colleagues began the first hiPSC-based clinical trial to treat wet age-related macular degeneration [29,30]. However, enhancing the research and clinical potential of hiPSCs will require the ability to modify (i.e., deletion, mutation, and insertion) their genome at precise locations. In this review, we discuss the current state of genome editing technologies in hiPSCs, with an emphasis on zinc-finger nuclease- (ZFN), transcription activator-like effector nuclease- (TALEN), and clustered regularly interspaced short palindromic repeats (CRISPR)/Cas9-based methods.

## 2. Gene Targeting by Homologous Recombination (HR)

Homologous recombination (HR) has been extensively used to introduce foreign genetic material as a means to generate ‘knock-in’ or ‘knock-out’ animals (namely mice) to study gene function and interrogate the mechanisms of human disease [31,32,33]. Methods for HR take advantage of the endogenous DNA repair mechanisms of a cell to alter or replace a specific genomic locus with engineered transduced homologous genetic sequence. Broadly speaking, such targeting events can result in the deletion, addition, or mutation of one or a long sequence of nucleotides. Traditional methods for HR-based gene targeting in pluripotent stem cells utilize electroporation, nucleofection, or chemical-based transfection methods to introduce linearized plasmid DNA constructs with homology arms of ~5 kb flanking positive selection cassettes (e.g., Blasticidin, Geneticin, Hygromycin B, Puromycin, Zeocin), resulting in disruption or deletion of one or more exons of the targeted gene. While isogenic DNA constructs were demonstrated to be a key factor for efficient targeting in mouse embryonic stem cells [34], several studies have demonstrated that HR in human cells is efficient with non-isogenic DNA [35,36,37]. However, the comparatively low frequency of homologous recombination compared to random insertion necessitates the need for the addition of a negative selection marker. Typically, the Herpes simplex virus thymidine kinase (HSV-tk) is inserted distally to one of the homology arms to increase the selection of clones with targeted gene insertions. With such a positive–negative selection system, randomly targeted cells will retain HSV-tk (and therefore be sensitive to the presence of Gancyclovir), while cells that are Gancyclovir-resistant will not have integrated the HSV-tk gene, potentially representing a HR event when occurring in the presence of the positive selection drug. In comparison to mouse pluripotent stem cells (mPSCs), such conventional methods for gene targeting applied in hPSCs are extremely inefficient, with targeting efficiencies varying between <0.1% and 5% [36,38,39,40,41]. Several reasons for the limited efficiency of HR in hPSCs compared to mPSCs have been postulated, including a poor rate of single-cell survival [42,43] as well as differences in DNA repair processes [44,45] and developmental stage [46,47].

## 3. Endogenous Repair of Double-Stranded DNA Breaks (DSB)

**Figure 1:**
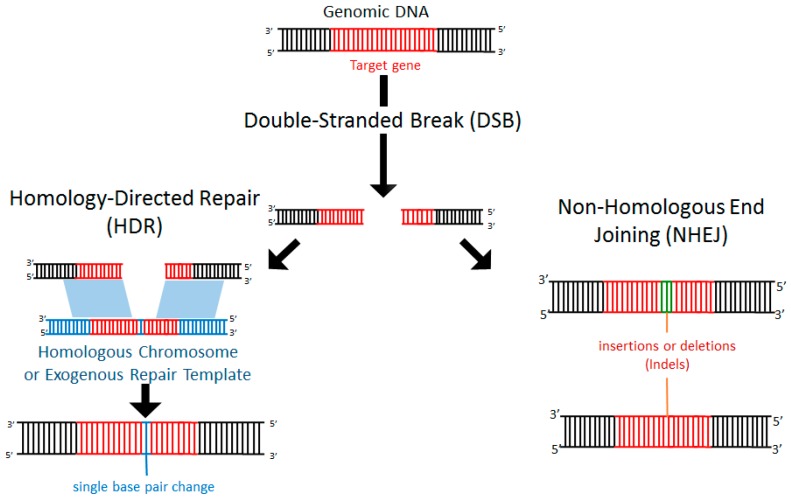
Double-stranded breaks (DSBs) induce endogenous DNA repair mechanisms. DSBs can be repaired by non-homologous end joining (NHEJ) or homology-directed repair (HDR). NHEJ often leads to deleterious insertions or deletions (indels), while HDR leads to high-fidelity DNA repair using the homologous chromosome or exogenously introduced DNA as a template.

Within a cell, DNA damage can occur due to endogenous replication errors or exogenous factors such as exposure to ionizing radiation, oxygen free radicals, or mechanical stress [48,49]. Exposure to such deleterious events is common and arises during normal cellular processes. Importantly, healthy cells are equipped with endogenous repair mechanisms to detect DNA damage and recruit machinery to the repair site [50]. In particular, following a double-stranded break (DSB), the cell can activate repair mechanisms through non-homologous end joining (NHEJ) or homology-directed repair (HDR). NHEJ is a process by which the cell can repair nucleotide mismatches or stand breaks without the need for a homologous template. Often NHEJ results in insertion or deletion of nucleotides (termed indels), resulting in disruption (e.g., frameshift mutations, premature stop codons, deletion) of the affected gene (Figure 1). By contrast, in HDR, a sequence of DNA that is partially homologous (typically the undamaged homologous chromosome) serves as a template for repair (Figure 1). Alternatively, if a DSB occurs in the presence of an exogenously introduced homologous repair template, the addition of specific DNA sequences can occur [51] (Figure 1).

Because it has been well established that DSB significantly enhance HR in human cells [52,53], significant effort has been focused on engineering nucleases that can induce DSB at targeted genomic locations. Two pioneering studies by Rouet and colleagues in the 1990s demonstrated that DSBs created by site-specific nucleases (SSNs) can be successfully repaired by NHEJ or through the endogenous homology-mediated mechanism using an exogenously provided repair template [54,55]. Later the same group showed that in mESCs a rare cutting endonuclease could induce site-specific DSBs, and such sites exhibited ‘two-sided’ homologous recombination, the first demonstration that targeted DSBs in pluripotent cells are recombinogenic [56]. Overall, these studies have served as a foundation for the development of designer nucleases, which will be discussed in the next section.

## 4. The Age of Designer Nucleases: ZFNs, TALENs, and CRISPR/Cas9

**Figure 2:**
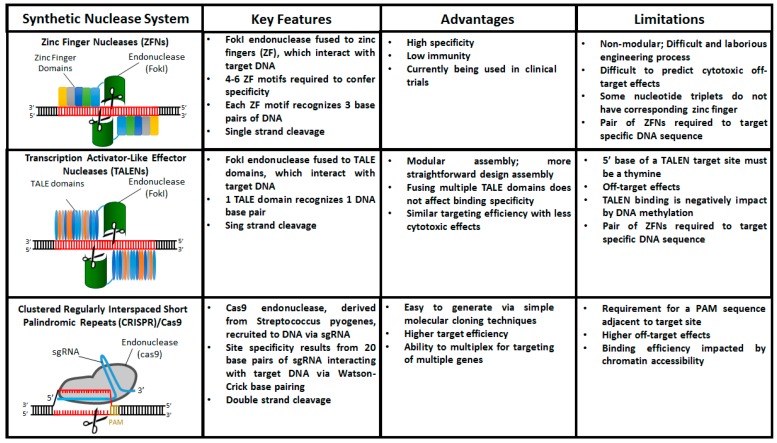
Comparison of engineered nucleases used for targeted gene editing in hiPSCs.

Over the past 15 years the three prominent genome editing tools that have emerged are zinc finger nucleases (ZFNs), transcription activator-like effector nuclease (TALEN), and clustered regularly interspaced short palindromic repeats (CRISPR)/Cas9. These technologies are similar in that they consist of two domains—a catalytic domain to initiate DSBs and a programmable domain which recognizes specific DNA sequences. In this section, we will discuss the basic principles of each of these technologies as well as their advantages and limitations (Figure 2).

### 4.1. Zinc Finger Nucleases (ZFNs)

Zinc finger nucleases (ZFNs) consist of a customized DNA binding domain and a DNA cleavage domain [57]. The DNA-binding domain contains a sequential fusion of multiple zinc finger domains, which bind to specific DNA target sequences [57]. Each zinc finger domain binds a three base pair sequence of DNA. As such, the DNA binding domains of ZFNs typically consist of an array of six linked zinc finger domains that confer specificity to a unique target sequence [58]. The DNA cleavage domain employs the type IIs restriction endonuclease FokI. Because the double-stranded cleavage activity of FokI requires dimerization, a pair of ZFNs that bind on opposite sides of the target site is used [59].

To date, there are many reports of ZFN-based genome editing of a variety of organisms, including human cells [57]. The first demonstration of a ZFN-targeted gene correction in human somatic cells was performed in 2003 by Proteus and Baltimore, where the authors used known ZFN recognition sites in a mutated GFP cassette to demonstrate successful gene correction and GFP expression [60]. More recently, the clinical application of ZFNs has rapidly advanced, including a Phase I clinical trial using ZFN-modified autologous CD4 T cells with a mutated CCR5 gene to treat HIV infection [61,62,63].

Despite the advances of ZFN-based methods in basic and translational research, ZFN technology has several limitations. First, because zinc finger domains have a higher affinity for G-rich sequences, there are several three-base nucleotide sequences that do not have associated zinc finger binding domains, which limits the robustness and specificity of ZFN-mediated cleavage [64]. Second, off-target cleavage by ZFNs can alter the function of unknown genes, introduce oncogenic translocations, and result in elevated cytotoxicity [65]. Finally, the generation of functional ZFNs requires a significant amount of expertise in protein engineering [66,67].

### 4.2. Transcription Activator-Like Effector Nucleases (TALENs)

Similar to ZFNs, transcription activator-like effector nucleases (TALENs) are a fusion protein consisting of nonspecific FokI endonuclease and an adaptable TALE DNA-binding domain. The TALE domains, derived from plant pathogenic bacteria [68], consist of a highly conserved sequence of 33–35 amino acid repeats with variable amino acids found at the 12th and 13th position [69,70]. The amino acids located at these positions, referred to as the repeat variable diresidues (RVD), confer sequence specificity to the TALENs. Specifically, there are four RVDs that contain the amino acid sequences Asn/Ile (NI), Asn/Asn (NN), Asn/Gly (NG), and His/Asp (HD), which recognize the nucleotides adenine, guanine, thymine, and cytosine, respectively [69,70]. Because of this one RVD to one base pair ratio, modular assembly methods can be used to engineer a series of TALE repeats that bind with high affinity to a single genomic locus [71,72]. Finally, similar to ZFNs, a pair of TALENs is required to target a specific stretch of DNA.

Overall, compared to ZFNs, TALENs have several advantages. Unlike ZFNs, in which the linking of multiple zinc finger domains can reduce DNA-binding specificity [73], fusing multiple TALE domains does not affect binding specificity [71,72,74]. Moreover, TALENs do not require the complex assembly processes that are required of ZFN construction [75]. In fact, high-throughput assembly methods [76] have allowed for the generation of a library of TALENs that span over 18,000 protein-coding genes in the human genome [77]. Additionally, compared to ZFNs, TALENs demonstrate similar targeting efficiency with less cytotoxic effects [78].

Even though TALEN-based methods have been used extensively to target numerous endogenous loci in human cells [72], there are still several shortcomings. Similar to ZFNs, deleterious undesired off-targets remain a concern with the use of TALENs [79]. Although TALENs do not have the same target-site restrictions that ZFNs have, the 5’ base of a TALEN target site must be a thymine [80], which may restrict applications where the targeting of specific single nucleotide is required. In addition, there are several reports that TALEN binding is negatively affected by DNA methylation [81], which may further impact successful targeting near gene regulatory regions, such as promoters, that are known to be highly methylated [82,83].

### 4.3. Clustered Regularly Interspaced Short Palindromic Repeats (CRISPR)/Cas9

Clustered regulatory interspaced short palindromic repeats (CRISPR) are adapted from the RNA-based immune system of prokaryotes, which confer resistance to bacteriophages through the CRISPR-associated protein (Cas) endonuclease-based destruction of foreign DNA [84,85]. As it relates to the in vitro targeting of genomic sequences in eukaryotic cells, the most common CRISPR/Cas system utilized is the Type II system from *Streptococcus pyogenes* [86,87]. The Type II CRISPR system consists of CRISPR associated protein 9 (Cas9) endonuclease and two short non-coding RNAs—a CRISPR (crRNA), which contains a seed sequence complimentary to the target DNA sequence (termed protospacer), and a transactivating crRNA (tracrRNA), which hybridizes with the crRNA to facilitate the endonuclease activity of the CRISPR/Cas9 complex [88]. When all three components are delivered to a target cell, a three base pair NGG protospacer-associated motif (PAM) on the target DNA strand guides Cas9 endonuclease to cleave three base pairs upstream of the PAM sequence [88,89,90]. The CRISPR/Cas9 system is highly adaptable as targeting Cas9 to a specific genomic locus can be achieved via cloning a 20 nucleotide sequence that is complementary to the protospacer into the crRNA construct. Moreover, to further facilitate the implementation of CRISPR/Cas9-based genome editing, more recent versions utilize a chimeric single-guide RNA (sgRNA), which consists of fused cRNA and tracrRNA [91].

Besides its simplicity and ease of use, the CRISPR/Cas9 system has several advantages over ZFN- and TALEN-based methods. Compared to TALENs, CRISPR/Cas9 exhibits lower cytotoxicity and higher targeting efficiencies [92]. Additionally, because of the small size and versatility of the sgRNA, several studies have demonstrated the simultaneous delivery of multiple sgRNAs to enable targeting of multiple genes [86,93]. Such multiplexed genome engineering strategies will greatly accelerate the study of the complex gene interactions that are the basis of multiple developmental programs and diseases.

Even though the CRISPR/Cas9 system is highly adaptable and easy to engineer, there are several concerns that may hinder its future applications. The requirement for a PAM sequence, which occurs roughly once every eight base pairs, may prevent the targeting of specific base pairs [94]. Another major impediment to the use of CRISPR/Cas9-based technologies for functional studies, as well as translational application, is the high frequency of off-target cleavage events [95,96,97]. An especially alarming study in human cells reported that off-target sites contained up to five mismatches and many off-target sites were mutated at frequencies comparable to or higher than target sites [96].

In order to overcome some of these limitations, especially with respect to off-target mutations and indel formation, several Cas9 variants have been engineered (Table 1). For example, several reports describe the generation of a Cas9 nickase (Cas9n), which generates a single-strand DNA break (SSB) rather than the DSB typical of wild-type Cas9 (spCas9) [86,98]. Compared to DSBs, the nicked DNA is predominately repaired through the high-fidelity HDR mechanism, using the uncut complementary strand as the template [99]. The use of Cas9n has been shown to reduce off-target effects while retaining the targeting efficiency of spCas9 [86,98]. Moreover, double-stranded nicking facilitated by dual sgRNA Cas9n pairs (Cas9dn) showed increased target specificity with a 50- to 1500-fold reduction of off-target indel formation [100]. Along similar lines, the use of fused catalytically inactive Cas9 (dCas9) and FokI nuclease (fCas9), which can induce DSBs through FokI dimerization, can modify target DNA sites with a higher specificity and similar efficiency to that of Cas9dn [101]. More recently, Howden and colleagues reported the fusion of spCas9 to a peptide derivative of the human Geminin protein (spCas9-Gem) [102], facilitating the degradation of Cas9 in the phase of cell cycle during which error-prone NHEJ primary occurs [103]. This programmable editing system was shown to significantly decrease NHEJ-induced indels at the target locus [102]. Finally, Komor and colleagues implemented an engineered Cas9n fused to a cytidine deaminase and uracil DNA glycosylase inhibitor programmed with a sgRNA to facilitate the direct base pair conversion of a cytosine to thymine or, guanine to adenine, within a five base pair window in the protospacer [104]. The use of this base editing (BE) technology was reported to facilitate the direct base conversion with 15%–75% efficiency in human cells without creating a DSB. As such, direct base conversion eliminates the possibility of NHEJ events and has numerous implications in disease modeling applications that target point mutations.

In addition to the common Cas9 endonuclease, Cpf1 is an RNA guided endonuclease derived from a class II (or type V) CRISPR system that has shown promise for gene editing applications in human cells [105]. Cpf1 differs from Cas9 in a few key ways including the requirement of only a single guide RNA without the need for a tracrRNA, single endonuclease domain, thymidine-rich PAM recognition sequences (e.g., 5’-TTTN-3’), and production of a 5-nt staggered DSB distal to the PAM sequence (in contrast with Cas9 which forms blunt end breaks) [106]. A recent analysis of Lachnospiraceae bacterium Cpf1 (LbCpf1) and Acidaminococcus sp. Cpf1 (AsCpf1) compared targeted editing efficiencies to spCas9 at ten chromosomal target sites that contain PAM sequences for both spCas9 and each Cpf1 variant. The study demonstrated that while spCas9 has the highest mutation rate (32% ± 4%), the LbCpf1 and AsCpf1 variants showed comparable mutation rates (19% ± 6% and 20% ± 5%, respectively) [105]. Although CRISPR-Cas Cpf1 technology is in its infancy, there are numerous advantages that may simplify multiplex gene editing. Multiplex gene editing using Cas9 nucleases require large constructs or multiple plasmid delivery. In contrast, Cpf1 alone is required for crRNA maturation where a single RNA pol III promoter can be used to drive expression of multiple crRNAs, allowing for targeting of up to four genes simultaneously in human cells [107].

### 4.4. Practical Considerations for Synthetic Nuclease Mediated HR

The use of synthetic nucleases in applications (see Section 5) that implement HDR requires the co-delivery of homology templates. Conventional approaches use a linearized or plasmid donor vector that contains ~10 kb (the upper limit of typical targeting vectors) of homologous DNA and an antibiotic resistance cassette. Still, such approaches are encumbered by low targeting efficiencies, even in the presence of a synthetic nuclease-induced DSB [108]. Because it has been reported that increasing the homology arms significantly increases targeting frequencies [109,110], bacteria artificial chromosomes (BACs) have been used as a method to create targeting vectors with homology arms in excess of 100 kb [111]. However, BAC-based methods require the use of recombineering technologies that may not be accessible to all researchers [112]. Alternatively, the delivery of homology templates via adeno-associated viruses (AAV) has been shown to facilitate improved gene targeting [113,114,115,116]. The mechanisms to explain this high targeting efficiency are not well understood, but it has been proposed that AAV is a single-stranded DNA virus and, upon infection and entry into the cell, this single-stranded piece mimics DNA damage and provides an ideal substrate for the endogenous DNA repair machinery, thereby significantly increasing gene-targeting efficiencies [117].

One primary concern with plasmid-, BAC-, and AAV-based methods for delivery of homology templates is that the identification of properly targeted cells requires the use of selection cassettes, which may affect neighboring gene expression and regulation [118,119]. To overcome this limitation, several groups have employed the Cre/loxP system to remove the selection transgenes after genome targeting [118,120]. With this system, selection cassettes flanked by two *loxP* sites are removed after Cre-mediated recombination [121]. Finally, the use of single-stranded oligonucleotides (ssODN), which are easily synthesized, offers a viable alternative for delivery of ‘scarless’ homology templates [122,123,124]. These ssODNs contain 60bp homology arms at a minimum and have been reported to more efficiently mediate SNP conversions via HDR [125,126,127,128] However, current ssODN-mediated methods still suffer from low efficiency (<1%), requiring high-throughput screening methods to identify targeted clones [122].

## 5. Applications of Gene Editing Technologies with hiPSCs

The various gene editing technologies discussed here have several applications as it relates to the research and clinical applications of hiPSCs. First, these methods can be used to engineer hiPSC lines with targeted reporter genes or selection markers. Such lines are useful to study the underlying mechanisms that regulate cell fate decisions, optimize protocols for differentiation into specific cells types, and isolate cell populations to be used in disease modeling and cell replacement therapies. Second, the generation of hiPSCs line with targeted mutations introduced into their genomes can be used to interrogate the onset and progression of various diseases. This is especially important with respect to the generation of isogenic hiPSCs as genetic and epigenetic variances between healthy control and disease lines may confound the interpretation of observed phenotypic differences. Finally, gene targeting strategies have important implications concerning the genetic modification of hiPSCs to be used in cell therapies. Although a discussion of all examples of these applications with hiPSCs is outside the scope of this review, we will discuss key examples in this section.

### 5.1. Generation of Targeted Reporter Lines

Several methods including chemical-based transfection reagents, electroporation, or viral infection have been successfully employed to randomly integrate reporter elements into the genome of hPSCs [128,129,130]. However, these approaches are hindered by poor characterization of regulatory elements known to control gene expression, positional effects that may lead to transgene silencing, and possible integration into sites that affect cell phenotype. On the other hand, the ability to insert reporter genes into specific genomic loci in hiPSCs offers the opportunity to faithfully reproduce patterns of gene expression with minimal effects on adjacent genes.

Several early studies reported the use of genome editing technologies to insert fluorescent proteins into loci associated with the undifferentiated state of hiPSCs [131,132,133]. For example, ZFNs [132], TALEN [131,134], and CRISPR/Cas9 [131,133] technologies have been used to insert green fluorescent protein (GFP) downstream of the last exon of OCT4. Importantly, not only did such lines faithfully recapitulate OCT4 expression to monitor hiPSC pluripotency, but also they did not impact the ability of targeted cells to differentiate into mature cell types, such as NKX6.1+SOX9+ pancreatic progenitor cells.

Targeted lines have been developed to monitor the differentiation of hiPSCs and isolate various, often rare, therapeutically relevant cell types. Forster et al. used ZFNs to generate an endogenous LGR5-GFP reporter line [135]. Through the FACS-based isolation of rare LGR5-GFP+ cells from differentiating cells, the authors were able to generate intestinal organoids with a composition and organization that mimicked those of in vivo intestinal tissue. ZFN-mediated methods have also been employed to generate GFAP-GFP astrocyte reporter lines from one healthy control and two ALS patient hiPSC lines [136]. Subsequent analysis of GFAP-GFP+ cells demonstrate their functional maturity and ability to engraft into a rat spinal cord, thereby paving the way for future cell-based therapies to treat ALS.

In the same vein, CRISPR/Cas9dn was used to generate a BAC-based homology template as a means to insert a GFP reporter before the stop codon of the PAX7, an early myogenic marker [137]. A similar approach was used to generate a fluorescent reporter line to mark MYF5+ cells, one of the earliest myogenic determination genes in the developing somites [138]. Collectively, these studies hold important implications for the prospective isolation of myogenic cells to be used in the modeling and treatment of muscular dystrophy.

Several groups have also used ZFN [139,140,141] and TALEN [142,143] approaches to introduce fluorescent proteins into safe harbor loci. In one important example, Wang and colleagues used ZFNs to introduce red fluorescent protein (RFP), luciferase, and herpes simplex virus thymidine kinase reporter constructs into the AAVS1 locus [141]. Remarkably, cardiomyocytes and endothelial cells derived from these targeted lines were successfully tracked in vivo using non-invasive bioluminescence imaging over the course of four weeks after being transplanted into mouse hearts. The ability to track the survival, engraftment, and location of hiPSC-derived cell populations in real time, in vivo is critically important for their clinical translation.

### 5.2. Using Designer Nucleases to Generate hiPSC-Based Disease Models

Broadly speaking two approaches can be used to generate isogenic disease models in hiPSCs with synthetic nuclease—the introduction of disease mutation into healthy control hiPSCs or the correction of the disease-related mutation in patient hiPSCs. In both approaches, applications in which the targeted gene will simply be rendered non-functional via frameshift indel mutations or partial deletions do not require the co-delivery of homology templates whereas applications in which a specific disease-related mutation will be corrected or introduced will also require the introduction of donor template (see Section 4.4). For the sake of simplicity, the examples discussed in this subsection have been divided according to the specific engineered nuclease that was implemented.

#### 5.2.1. ZFN

Two early examples of ZFN-generated hiPSC models relate to the study of sickle cell disease [144,145]. In both examples, the authors elegantly used ZFNs templates to precisely correct mutations in hiPSCs derived from patients with mutated β-globin alleles. ZFNs have also been extensively used in generating hiPSC-based models of neurodegenerative diseases [127,146,147,148]. In particular, Soldner and colleagues used ZFN approaches with both plasmid and ssODN donor templates to introduce Parkinson’s disease (PD) mutations into wild-type cells as well as to correct the same mutations in diseased hiPSCs [127]. In a related study, the phenotypic analysis of midbrain dopaminergic neurons (the neuronal subtype predominantly affected in PD) derived from these isogenic lines revealed altered transcriptional network activity related to mitochondrial dysfunction observed in PD hiPSC-derived neurons [146]. Moreover, the authors used a small-molecule high-throughput screening approach with these isogenic hiPSCs to identify isoxazole as a potential therapeutic compound. In another example, genome-wide expression analysis of motor neurons (the neuronal subtype predominantly affected in amyotrophic lateral sclerosis (ALS)) derived from ZFN-corrected SOD1 mutant hiPSCs revealed a unique, disease-dependent transcriptional signature indicative of increased oxidative stress, reduced mitochondrial function, altered subcellular transport, and activation of the ER stress [148]. Finally, ZFN-mediated gene editing was used to develop two sets of isogenic hiPSC lines—one with a TAU-A152T mutation corrected, and another with the same homozygous mutation introduced. Because tauopathies are characteristic of numerous neurodegenerative disorders including Alzheimer’s disease (AD), frontotemporal dementia (FTD), and progressive supranuclear palsy (PSP) [149,150,151], neurons derived from these hiPSCs will be useful for the study of disease mechanisms and evaluation of therapeutic compounds.

#### 5.2.2. TALENs

Several groups have employed TALEN technologies to generate numerous hiPSC-models of disease including Duchenne muscular dystrophy [152]. Niemann–Pick Type C disease [153], Lesch–Nyhan syndrome [154], X-linked severe combined immunodeficiency [155], and various blood disorders [156,157,158,159]. One of the more well-designed applications of TALEN methods with hiPSC-based models was performed by Woodruff and colleagues to investigate the mechanisms by which mutations in presenilin-1 (PS1) contribute to familial Alzheimer’s disease (FAD) [160]. In this study the authors generated an allelic series of isogenic PS1 ΔE9 mutations, (WT/null, WT/ΔE9, ΔE9/ΔE9, ΔE9/null) in hiPSCs derived from J Craig Venter, a completely sequenced genomic background [161,162,163]. Phenotypic analysis of neurons generated from these isogenic lines revealed for the first time that FAD PS1 mutations do not act as through loss-of-function but rather exhibit gain of toxic activity. In a more recent study with these same cell lines, the authors determined that neurons with PS1 ΔE9 mutations displayed defective transcytosis of lipoproteins [163]. This novel finding may shed new light onto the mechanisms by which FAD mutations lead to neuronal degeneration in patients as neurons require extracellular cholesterol uptake from lipoprotein particles for axon elongation as well as synapse formation and maintenance [164,165,166].

#### 5.2.3. CRISPR/Cas9

Despite being in its infancy CRISPR/Cas9 technology has been widely applied to generate hiPSC-based models of human disease. Similar to the other gene editing technologies discussed here, CRISPR/Cas9 methods have been extensively applied in hiPSC to develop models of immunodeficiency and blood diseases including β-Thalssemia [167,168], hemophilia [169], ICF syndrome [170], sickle cell disease [171], and severe combined immunodeficiency [172]. More recently, CRISPR/Cas9 techniques have been combined with advanced bioengineering culture techniques to create more accurate in vitro disease models [173,174]. For example, Firth et al. used an air–liquid interface culture system to generate complex, multicellular pulmonary epithelial cell cultures from hiPSCs derived from patients with cystic fibrosis (CF) [173]. Remarkably, the correction of CF-related mutations in these cultures via CRISPR/Cas9 resulted in the restoration of functional expression of CFTR protein and chloride channel function. In another example, CRISPR/Cas9 genome editing coupled with engineered 'heart-on-chip' methods, were employed to elucidate the pathological mechanisms related to the cardiomyopathy of Barth syndrome (BTHS) [174]. Specifically, the authors were able to use cardiomyocytes derived from isogenic sets of hiPSCs to directly link the metabolic, structural and functional phenotypes associated with Barth syndrome to mutation of the gene encoding tafazzin. Finally, in a pioneering study by Howden and colleagues, the authors implemented CRISPR/Cas9 systems to simultaneously reprogram and correct the disease-related mutation in fibroblasts from a patient with retinitis pigmentosa [175]. This ability to generate isogenic cell lines quickly, efficiently, and without the need for drug selection will significantly advance the personalized medicine application of hiPSCs.

### 5.3. Development of Gene Edited hiPSCs for Cell-Based Therapies

The use of genome editing technologies with hiPSCs for regenerative medicine applications is rapidly accelerating. In one early example of such an approach, Yusa and colleagues used a ZFN approach for the bi-allelic targeted correction of a point mutation in the α1-antitrypsin (A1AT) gene that results in an α1-antitrypsin deficiency (A1ATD), the most common inherited liver metabolic disorder [176]. Remarkably, when these corrected hiPSCs were differentiated to hepatocyte-like cells and injected into a mouse model of liver disease, the cells not only incorporated into in vivo liver tissue but displayed functional activities, such as long-term secretion of human albumin. As such, this approach has great potential for the treatment of numerous genetic liver disease, for which the only current therapy is liver transplantation.

Based on the revolutionary studies that demonstrated a patient, the so-called ‘Berlin Patient’, had been successfully cured of HIV infection following allogeneic hematopoietic stem cell (HSCs) transplants from a homozygous CCR5Δ32 donor [177], there has been significant effort in generating similar cell types from hiPSCs [178]. To that end, Ye et al. used both TALEN- and CRISPR/Cas9-based methods to introduce bi-allelic CCR5Δ32 into wild-type hiPSCs [179]. Moreover, monocytes and macrophages differentiated from these mutated hiPSCs were resistant to in vitro HIV-1 infection. Overall, this approach has great promise to provide an unlimited source of HSCs as a means to functionally cure HIV patients.

## 6. Future Perspectives

As gene editing technologies continue to evolve, there are several emerging trends relating to their use in hiPSC-based efforts in disease modeling, drug discovery, and regenerative medicine. For example, in applications that require the use of HDR (see Section 4.4), the low targeting efficiencies remain problematic. To address this challenge, Yu et al. implemented a high-throughput screening approach to identify small molecules to enhance CRISPR/Cas9 genome editing in pluripotent stem cells [180]. The authors discovered that two small molecules, L755507 and Brefeldin A, could improve CRISPR/Cas9-facilitated HDR efficiency (3-fold for large DNA insertions and 9-fold for point mutations). In addition, the authors identified another compound, azidothymidine, that increased indel mutations mediated by NHEJ through the inhibition of HDR. Along similar lines, several studies have shown that epigenetic modifications, such as DNA and histone modifications, may negatively impact TALEN [81] and CRISPR/Cas9 [181]. The use of epigenetic modifiers such as histone deacetylases and DNA methyltransferases in conjunction with genome editing tools has shown promise to address such issues [81].

Another future application of the genome editing technologies discussed in this review relates to the immunogenicity of hiPSCs. Much of the excitement surrounding the development of hiPSC-based technologies was with regard to the potential of these cells to serve as an unlimited supply of autologous cells for cell-based therapies without the concerns about immune rejection that plagued the use of hESCs. However, there have been several conflicting reports regarding the immune tolerance of hiPSCs and their derivatives, with some reports suggesting that hiPSCs are completely immune privileged and others suggesting that hiPSCs are highly immunogenic [182,183,184,185,186]. One potential approach to address these immunological-related issues that has been previously applied to hESCs [187] is the use of gene editing technologies to introduce specific genes into safe harbor loci that confer immune protection to hiPSCs and their derivatives.

At the pace at which the genome editing field has been advancing over the past few years, it is likely that new technologies with characteristics superior to the methods discussed in this review are likely to emerge. For example, a novel method that employs a DNA-guided endonuclease derived from the *Natronobacterium gregoryi* Argonaute (NgAgo) has been shown to be advantageous in Cas9 methods as it does not require the presence of a PAM sequence and has fewer off-target effects [188,189]. However, this technology has yet to be applied to hiPSCs and there are concerns about its reproducibility for genome editing in human cells [190,191,192].

Finally, the use of gene-edited hiPSCs in basic science and translational applications will require overcoming additional technological bottlenecks unrelated to gene editing technologies per se. For example, issues related to clonal variation, the heterogeneity and impurity of differentiated cell populations, and scalable production methods [193,194,195,196,197] need to be overcome, which may limit the future application of hiPSCs in disease modeling, drug discovery, and cell-based therapies.

## Figures and Tables

**Table 1:** Summary of engineered Cas9 variants for gene editing applications.

Cas9 Variants	Advantages	Disadvantages
Wild type *Streptococcus pyogenes* Cas9 (spCas9)	Programmed RNA guided editing; High specificity; Easily engineered	dsDNA breaks repaired by NHEJ forming indels
Cas9 nickase (Cas9n)	No dsDNA break induced; Promotes homology directed repair (HDR)	Some nicks go through a dsDNA break intermediate that can be repaired by NHEJ
Dual sg-RNA-Cas9 nickases (Cas9dn)	Increased specificity, dual sgRNA, promotes higher HDR over single nickase.	Must design dual sg-RNA-Cas9n complexes targeting opposite DNA strands
Cytidine deaminase fused Cas9 (D10A)	No dsDNA break induced; Increased efficiency over spCas9; Direct base conversion of C→T	Five base pair editing window; specific C→T conversion
spCas9-Gem	Regulates Cas9 presence at each stage of the cell cycle; Efficiently generates knock-in reporter lines and gene correction	Decreases frequency of NHEJ indels at target locus

Abbreviations: DSB = double-stranded DNA break; NHEJ = Non-homologous end joining; HDR = Homology directed repair.
